# Diabetic Pleurisies

**Published:** 1911-10

**Authors:** 


### Diabetic Pleurisies

Braillon, *Rev. de Med.,* April 10, 1911, seems to be the first to have made a thorough study of this subject since its inception by Dieulafoy and Ramond. Putrid pleurisies are due to pulmonary gangrene. Sero-fibrinous forms usually develop after the fiftieth year, in cases of moderate severity, without emaciation, corresponding to the clinical type of arthritic diabetes. Arterial supertension, ventricular hypertrophy, bruit de galop and albuminuria are usually found. Dsypnoea is disproportionate to the moderate accumulation of fluid.

